# The Relationship Between Depression and Anxiety Symptoms of Adult PWE and Caregivers in a Tertiary Center

**DOI:** 10.3389/fneur.2022.766009

**Published:** 2022-03-09

**Authors:** Rafael Batista João, Mateus Henrique Nogueira, Márcia Elisabete Morita-Sherman, Marina Koutsodontis Machado Alvim, Steven Johnny, Haryton Pereira, Hildete Prisco Pinheiro, Fernando Cendes, Clarissa Lin Yasuda

**Affiliations:** ^1^Neuroimaging Laboratory, Department of Neurology, University of Campinas, Campinas, Brazil; ^2^The Brazilian Institute of Neuroscience and Neurotechnology, University of Campinas, Campinas, Brazil; ^3^Institute of Mathematics, Statistics and Scientific Computing, University of Campinas, Campinas, Brazil

**Keywords:** epilepsy, caregivers, depression, anxiety, suicidality

## Abstract

**Background:**

Although several studies have emphasized the association between epilepsy and psychiatric disorders, fewer have investigated the impact of epilepsy on caregivers' emotional status, mainly in adult people with epilepsy (PWE). Here we investigated depressive symptoms, suicidal ideation, and anxiety symptoms in a large group of adult PWE and their caregivers.

**Methods:**

We analyzed symptoms of depression [with the Beck Depression Inventory-II (BDI-II)], suicidal ideation (with BDI-II item 9), and anxiety symptoms (with the Beck Anxiety Inventory) in a large group of adult PWE [*N* = 548 (60% women; median age 41)] and caregivers [*N* = 191 (72% women; median age 47)] from a Brazilian tertiary center, considering sociodemographic and clinical aspects. We also applied the Liverpool Adverse Events Profile to assess anti-seizure drugs adverse events.

**Results:**

While the presence (*p* = 0.026) (and intensity, *p* = 0.007) of depressive symptoms and suicidal ideation (*p* = 0.02) were higher in PWE compared to caregivers, the proportion of clinical anxiety symptoms (*p* = 0.32) (and the intensity, *p* = 0.13) was similar in both groups. Although the rates of suicidal ideation were higher in focal epilepsy (20%), both generalized genetic epilepsy and caregivers also presented elevated frequencies (11%) of suicidal ideation. The analyses of 120 patient-caregiver dyads revealed that the intensity of depressive symptoms in PWE (but not anxiety) correlated with the intensity of depressive (*r* = 0.35; *p* < 0.001) and anxiety (*r* = 0.25; *p* = 0.01) symptoms in their caregivers. In the multivariate analyses of PWE, focal epilepsy (compared to GGE) was associated with clinical depressive symptoms (odds ratio, OR 2.1) and suicidal ideation (OR 3.2), while recurrent seizures (compared to the seizure-free group) were associated with suicidal ideation (OR 2.6) and anxiety symptoms (OR 2.1). Also, caregivers with anxiety symptoms were 8 times more likely to exhibit depressive symptoms, and those with depressive symptoms were 8 times more likely to present anxiety symptoms.

**Conclusion:**

Our study suggests that specific attention for the caregivers' mental health is as essential as PWE. There is an urgent need for more studies involving caregivers to identify their emotional distress and provide adequate treatment.

## Introduction

The impact of epilepsies extends beyond recurrent seizures and their consequences, such as falls, accidents, and fractures (1). Epilepsies are highly associated with cognitive dysfunction (2), mood disorders, a higher risk of suicidal ideation and other psychiatric abnormalities (3, 4). The multifactorial characteristic of the poor quality of life in people with epilepsy (PWE) yields a great challenge to be addressed by physicians, health professionals, and caregivers (4).

While several studies have investigated cognitive dysfunction and psychiatric abnormalities in PWE, less attention has been directed to the impact of epilepsy on the emotional status of relatives and caregivers (5, 6). The caregivers of PWE are involved with support strategies, such as medication management, frequent visits to health care centers and help with accidents related to seizures. This intense demand may lead these individuals to chronic stress and emotional coping difficulties (6).

Many studies have evaluated caregivers' emotional distress of other chronic diseases (7–9); however, fewer have investigated depressive and anxiety symptoms in caregivers of PWE (especially in adults). We hypothesized that the unpredictability of seizures and elevated risk of accidents (and sudden death) affect PWE's mental health, their families and caregivers (10). Several studies evaluated the burden on family caregivers of children with epilepsy. Parental emotional distress is a well-known condition in this context, as those patients may demand chronic and intensive care (11, 12). Although the caregivers of the pediatric population have been evaluated in epileptology, the emotional status of caregivers of adult PWE is still poorly understood. We believe this is of extreme importance, especially in developing countries such as Brazil, in which adult PWE have a high frequency of psychiatric manifestations (13). Therefore, we aimed to investigate the occurrence of depression symptoms, suicidal ideation, and anxiety symptoms in a large group of PWE and caregivers and analyze demographic and clinical aspects associated with these symptoms.

We tested the following hypotheses:

1. Caregivers may present depressive and anxiety symptoms as the adult PWE they follow.
2. The severities of depression and anxiety symptoms are positively correlated between PWE and their related caregivers.
3. The pattern of seizure control may affect the severity of depressive and anxiety symptoms of their caregivers.

## Materials and Methods

### Subjects Selection

We evaluated 739 consecutive subjects between 2016 and 2017 (548 non-institutionalized PWE and 191 caregivers) currently followed at our outpatient epilepsy clinic (Tertiary hospital at the University of Campinas, UNICAMP, São Paulo, Brazil) with interviews and questionnaires to investigate depression, suicidal ideation, and anxiety symptoms.

We divided patients into focal epilepsy [417 subjects, (252 women), median age 43, range 18–83 years], genetic generalized epilepsy [GGE = 74, (48 women), median age 33, range 18–60 years], and unknown epilepsy [UE = 57 subjects, (27 women), median age 38, range 18–65 years].

We included a large sample of PWE caregivers [191 subjects, (137 women), median age 47, range 18–82]. The group of caregivers included relatives (genetically related and unrelated) and non-relatives who live in the same environment as the patients. These caregivers were in close contact with their respective patients and were responsible for helping them with medications, consultations, seizures, and daily life problems. Among those 191 caregivers, we obtained paired data from 120 dyads (PWE and their respective caregivers) collected on the same day of consultation. None of the caregivers were private health professionals, and only two patients presented mild developmental delays.

### Clinical and Sociodemographic Data

Patients and caregivers were assessed on the day of the medical appointment. Clinical and sociodemographic data were collected during the interview and from medical charts. Clinical data included epilepsy type (focal, genetic generalized epilepsy, and unknown epilepsy), seizure control (recurrent seizures, fluctuating, and seizure-free) (14), anti-seizure drugs (ASD), depression and anxiety symptoms, and suicidal ideation. We also collected age, gender, employment status, marital status, and years of education. The local Ethics Committee approved this study, and all subjects signed a consent form to participate (Research Ethical Committee Number: 06816819.5.0000.5404).

### Psychiatric Symptoms and Anti-seizure Drugs Assessment and Instruments

We addressed the volunteers who accepted to participate in the study to an appropriate place to fill out self-administered scales (average duration of 30 min) under the supervision of undergraduate students, trained and previously monitored by a psychologist (M.H.N.). All participants were informed that non-participation would not influence the treatment of their respective patients.

To assess symptoms of depression, we applied the Beck Depression Inventory-II (BDI-II), a self-assessment scale used for screening and severity quantification of depressive symptoms (15). The BDI-II cut-offs for the Brazilian population were applied (0–13: subclinical depression, 14–19: mild depression, 20–28: moderate depression, and 29–63: severe depression), wherein PWE and caregivers with scores higher than 14 (16) were classified with clinical depressive symptoms. We used item nine of the BDI-II to evaluate suicidal ideation. A score equal to or >1 was set for the presence of suicidal ideation, based on studies that suggested this classification for assessing long-term vulnerability for suicide (17). We used the Beck Anxiety Inventory (BAI), a self-report scale to screen anxiety symptoms (18). Although the minimum cut-off for clinical anxiety is 11 (0–10: subclinical anxiety, 11–19: mild anxiety, 20–30: moderate anxiety, and 31–63: severe anxiety), we set the clinical anxiety scores as ≥14 to prevent false positives and provide a more balanced sensitivity and specificity. Accordingly, PWE and caregivers with scores higher than 14 were considered significant for clinical anxiety symptoms.

PWE also answered the Liverpool Adverse Events Profile (LAEP), an epilepsy-specific self-administered questionnaire with 19 items. The LAEP has a Likert scale with global scores ranging from 19 to 76. Scores ≥ 46 were considered significant for adverse events (19).

### Statistical Analysis

We used the Statistical Package for the Social Sciences—SPSS22 (Armonk, NY, USA) to perform statistical analysis. Categorical variables, expressed in percentages, were analyzed with the Chi-square test (*post-hoc* analyses with Bonferroni adjustment were applied for group comparisons) (20). The Kolmogorov-Smirnov test was performed to evaluate data distribution. Kruskal-Wallis tests were applied to compare continuous variables with non-normal distribution. Correlations between continuous non-normal distributed variables were assessed with Spearman tests. We also performed logistic regression models with clinical and sociodemographic variables to investigate factors associated with depressive, suicidal ideation, and anxiety symptoms. The significance level for the analyses was set at *p* < 0.05.

## Results

### Demographic and Clinical Data (PWE and Caregivers)

As showed in Table 1, PWE were younger than caregivers (*p* < 0.001) and presented higher rates of unemployment (*p* = 0.001) and non-married subjects (*p* < 0.001). We found a higher proportion (*p* = 0.004) of women in the caregiver's groups than PWE. Years of education were similar between the two groups (*p* = 0.9). While the proportion of depression symptoms (*p* = 0.026), the intensity of depressive symptoms (*p* = 0.007*)* and suicidal ideation frequency (*p* = 0.02) were higher in PWE compared to caregivers, the proportion of clinical anxiety (*p* = 0.32) and the intensity of anxiety symptoms (*p* = 0.13) were similar in both groups. We observed a similar proportion of concurrent clinical depression and anxiety in both groups (*p* = 0.23), with comparable intensity.

**Table 1:** Sociodemographic characteristics and clinical data (PWE and caregivers).

	**PWE ***N*** = 548 Median (range) or *N* (%)**	**Caregivers ***N*** = 191 Median (range) or *N* (%)**	***p*** **-value**
Median age	41 (18–83)	47 (18–82)	<0.001
**Gender**
Women	327 (60%)	137 (72%)	0.004
Men	221 (40%)	54 (28%)	
**Employment status**
Unemployment	337 (62%)	92 (48%)	0.002
Employment	211 (38%)	99 (52%)	
**Marital status**
Married	228 (42%)	111 (58%)	<0.001
Non-married	320 (58%)	80 (42%)	
Years of education	11 (0–18)	10 (0–18)	0.9
**Clinical depression**
*N*	497	174	
Yes	207 (42%)	55 (32%)	0.026
No	290 (58%)	119 (68%)	
BDI-II score	11 (0–57)	7 (0–56)	0.007
**Suicidal ideation**
*N*	534	186	
Yes	99 (19%)	20 (11%)	0.02
No	435 (81%)	166 (89%)	
**Clinical anxiety**
*N*	492	168	
Yes	184 (37%)	55 (33%)	0.32
No	308 (63%)	113 (67%)	
BAI score	9 (0–58)	7 (0–51)	0.13
**Concurrent clinical depression and anxiety**
*N*	457	160	
Yes	124 (27%)	35 (22%)	0.23
BDI-II score	25 (14–57)	24 (14–56)	0.65
BAI score	25 (14–56)	29 (15–51)	0.56

*PWE, people with epilepsy; BDI-II, beck depression inventory-II; BAI, beck anxiety inventory; p, p-value for pearson χ^2^-test of association between categorical variables and for Mann-Whitney test of comparison of medians for quantitative variables*.

### Caregivers' Analyses

Most caregivers with depressive symptoms were women (87% in the subgroup with clinical depression symptoms vs. 65% with non-clinical symptoms, *p* < 0.01). Similarly, most caregivers with anxiety symptoms were women (86% in the subgroup with clinical anxiety symptoms vs. 65% with non-clinical symptoms, *p* < 0.01). Considering the caregivers with combined anxiety and depressive symptoms, we observed that the majority were women (*p* = 0.002) and presented familiar antecedents of psychiatric disorders (39% in the subgroup with combined symptoms vs. 15% in the subgroup without combined symptoms, *p* < 0.01).

We obtained paired data from a subset of 120 patient caregivers' dyads, collected on the same consultation day. From this group of caregivers, 87 individuals were genetically related to PWE (first or second-degree relatives), and 33 were genetically unrelated. Symptoms of depression tended to be more frequent in genetically related (28%) than in genetically unrelated (23%) caregivers, although without statistical significance (*p* = 0.75). However, the presence of anxiety symptoms was similar in genetically related (29%) and unrelated caregivers (31%) (*p* = 1). We observed that the intensity of depressive symptoms in PWE (but not anxiety) correlated with the intensity of depressive (*r* = 0.35; *p* < 0.001) and anxiety (*r* = 0.25; *p* = 0.01) symptoms in their caregivers. While we identified higher LAEP scores in the PWE of caregivers with symptoms of depression (*p* = 0.026) and those with concurrent anxiety and depression (*p* = 0.038), neither the type of epilepsy nor the seizure control impacted their correspondent caregivers' frequency of anxiety and depression symptoms (Supplementary Table 1).

### Depression, Suicidal Ideation, and Anxiety Symptoms [PWE (Groups) and Caregivers] According to Epilepsy Types

As showed in Supplementary Table 2, PWE with GGE were younger (*p* < 0.001) than those with focal epilepsy and caregivers. Furthermore, the *post-hoc* analyses (with Bonferroni correction) showed a higher frequency of non-married subjects in the GGE group (*p* < 0.001) and married subjects among caregivers (*p* < 0.001). In addition, we observed increased unemployment rates among patients with focal epilepsy (*p* < 0.001) and equivalent years of education across the three groups (*p* = 0.1).

The presence (*p* = 0.026) and the severity (*p* = 0.018) of depressive symptoms was higher in the focal epilepsy group (51% with temporal lobe epilepsy) than the caregivers. Subjects with focal epilepsy presented more suicidal ideation than the GGE and caregivers' groups (*p* = 0.006). However, the three groups presented similar frequency (*p* = 0.56) and intensity (*p* = 0.3) of anxiety symptoms. We observed an equivalent proportion (~25%) of subjects with concurrent clinical depression and anxiety symptoms in the three groups (*p* = 0.38). In addition, the frequency (*p* = 0.8) and the intensity (*p* = 0.37) of ASD adverse events were equivalents between patients with FE and GGE.

**Figure 1:**
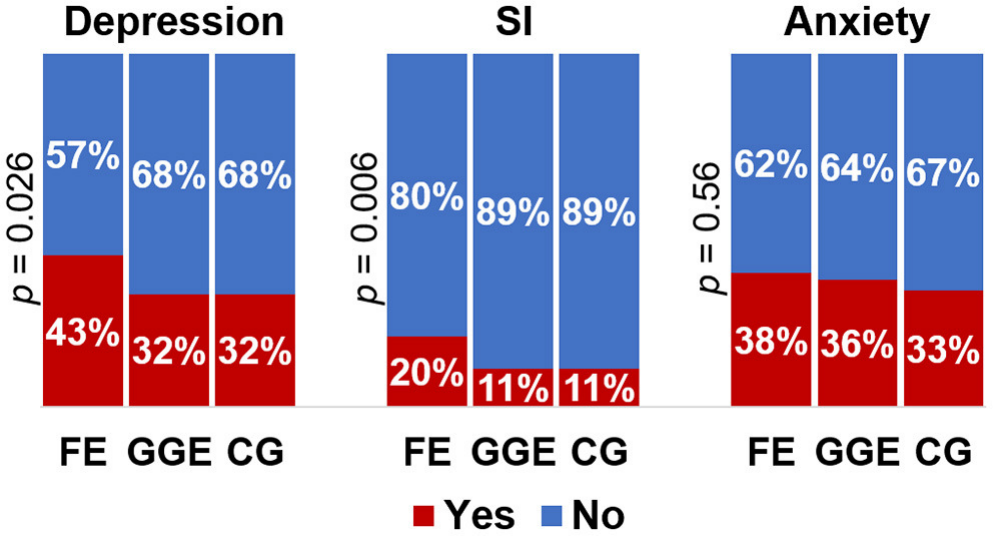
Distribution of depressive symptoms, suicidal ideation and anxiety clinical symptoms according to epilepsy type and caregivers. Depression, clinical depression symptoms; SI, suicidal ideation; Anxiety, clinical anxiety symptoms; FE, focal epilepsy; GGE, genetic generalized epilepsy; CG, caregiver; *p, p*-value for Pearson χ^2^-test of association between categorical variables.

The distributions of depressive, suicidal ideation, and anxiety clinical symptoms among the three groups according to epilepsy types are shown in Figure 1.

### Depressive Symptoms, Suicidal Ideation, Anxiety Symptoms, and ASD Adverse Events in PWE According to the Seizure Control

PWE were classified according to their seizure-control pattern as recurrent seizures, fluctuating, and seizure-free. The pairwise comparisons revealed that the recurrent-seizures group included a higher proportion of subjects with depression symptoms (compared to the seizure-free group; *p* < 0.01) and with suicidal ideation (compared to the fluctuating and seizure-free groups; *p* < 0.001). Moreover, the group with recurrent seizures also presented increased severity of depressive [compared to both fluctuating (*p* = 0.03) and seizure-free groups (*p* < 0.001)] and anxiety symptoms [compared to the seizure-free group (*p* < 0.001)]. We also observed more frequent ASD adverse events in the recurrent-seizures group (34%) when compared to the seizure-free (16%; *p* < 0.001). The severity of ASD adverse events (LAEP score) was higher in the recurrent-seizures group compared to the seizure-free group (*p* < 0.001; Supplementary Table 3).

**Figure 2:**
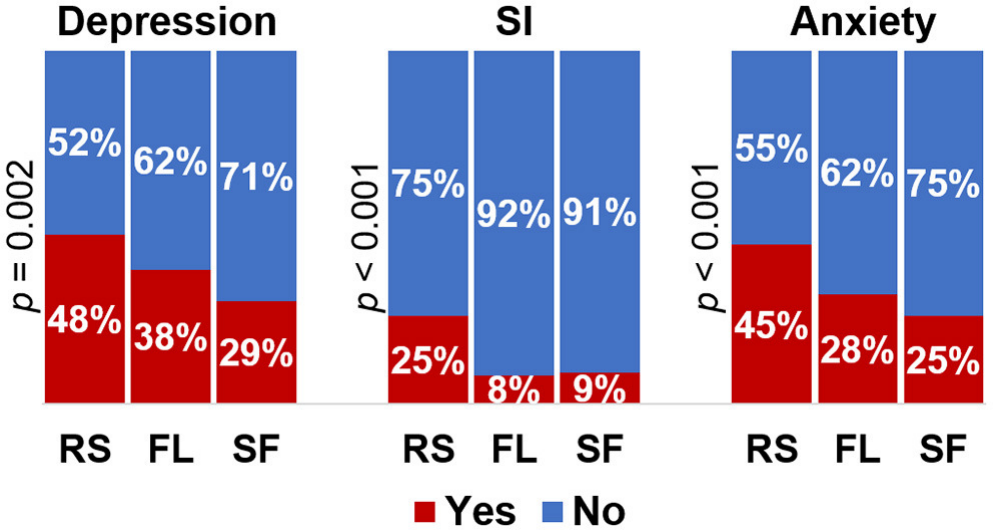
Proportions of depressive symptoms, suicidal ideation and anxiety clinical symptoms according to seizure control. Depression, clinical depression symptoms; SI, suicidal ideation; Anxiety, clinical anxiety symptoms; RS, recurrent seizures; FL, fluctuating; SF, seizure-free; *p, p*-value for pearson χ^2^-test of association between categorical variables.

The distributions of depressive, suicidal ideation, and anxiety clinical symptoms among the three groups according to the seizure control are shown in Figure 2.

### Factors Associated With Depressive, Suicidal Ideation, and Anxiety Symptoms in Adult PWE

We applied logistic regression to identify predictive factors associated with depressive, suicidal ideation, and anxiety symptoms in PWE. The predictor variables were the types of epilepsy (focal and GGE), sex, education, employment status, seizure control, and presence of depressive or anxiety symptoms (when appropriate). For depressive symptoms, the entire model explained between 24.2% (Cox and Snell *R*^2^) and 32.6% (Nagelkerke *R*^2^) of the variance, with an accurate overall prediction of 75.5% of the cases; it yielded an accurate prediction of 64.5% of the PWE with clinical depression. Anxiety symptoms and the type of epilepsy made a unique contribution to the model. The strongest predictor of depression symptoms was the presence of anxiety symptoms (odds ratio, OR 8) when controlled for other variables in the model. The second predictor of depressive symptoms was focal epilepsy with an OR of 2.1 (when controlled for the other variables included). Women with epilepsy were 1.7 folds more likely to present depressive symptoms than men (Supplementary Table 4).

Our model to assess predictors of suicidal ideation explained between 15.7 and 25.4% of the variance in suicidal ideation. Overall, it correctly classified the outcome for 81%; however, only 15.6% of the predictions for the PWE with suicidal ideation were accurate. After controlling for the variables in the model, the significant predictors were clinical anxiety symptoms (OR 5.23), focal epilepsy (OR 3.16, compared to GGE), and recurrent seizures (OR 2.57, compared to the seizure-free group). The increase of 1 year of age associated with a decrease in the odds of presenting suicidal ideation by a factor of 0.98 (Supplementary Table 5).

The model with predictors for anxiety symptoms accounted for between 26.1 and 35.6% of the variance in anxiety symptoms, with overall correct discrimination of the outcome for 76.6% (it accurately predicted 67.5% of the PWE with clinical anxiety). The strongest predictor of anxiety was the presence of depressive symptoms (OR 8) when controlled for other variables in the model. Recurrent seizures yielded an OR of 2.1 (compared to the seizure-free group), and women presented anxiety symptoms twice as much as men when controlled for other variables (Supplementary Table 6).

We also investigated predictors for comorbid anxiety and depression with a model that included the adverse effects (Supplementary Table 7). The model explained between 32 and 47% of the variance in the combination of anxiety and depression symptoms, with correct identification of the outcome in 85.4% (it precisely identified 72.6% of individuals with comorbid symptoms). After controlling for the variables in the model, the presence of adverse effects resulted in an OR of 19.8, while women were approximately twice more likely to present comorbid symptoms than men.

### Factors Associated With Depressive and Anxiety Symptoms in Caregivers of Adult PWE

We used logistic regression to identify predictor variables related to depressive, suicidal ideation, and anxiety symptoms in caregivers of adult PWE. The models included age, sex, education, marital status, and the presence of depressive or anxiety symptoms (when appropriate) as predictor variables. The model for depressive symptoms correctly discriminated the outcome for 75.5% of the cases (it accurately predicted 65.8% of the caregivers with clinical depression and 82% of those without). It explained between 22 (Cox and Snell *R*^2^) and 30% (Nagelkerke *R*^2^) of the variance in the depression of caregivers. The strongest predictor of presenting depression symptoms was clinical anxiety, with an OR of 8; women were 1.6 times more likely to have depression symptoms (compared to men) after controlling for the other variables in the equation (Supplementary Table 8).

Although the whole model with predictors of suicidal ideation was significant (chi-square = 56.7, df = 5, *p* < 0.001) and yielded an overall prediction of 81.5%, it was unable to accurately predict suicidal ideation in the group of caregivers, on the contrary, it successfully predicted the absence of suicidal ideation in 100%. Nevertheless, the strongest predictor for suicidal ideation was the presence of anxiety symptoms (OR 5.9), after controlling for the variables in the model. The coefficients showed that an increase of 1 year of age associated with a decrease in the odds of suicidal ideation by a factor of 0.98.

The model with predictors of anxiety symptoms accounted for between 24.5 and 33% of the anxiety variance and correctly classified the outcome for 75.9% of the cases. It accurately predicted the presence of anxiety symptoms in 68.4%. The coefficients revealed that the strongest predictor was the presence of depression symptoms with an OR of 8.4 after controlling for the other variables in the model. It also showed that women were twice more likely to present symptoms of depression compared to men; the non-married individuals were 1.7 times more likely to present depression than those who were married (Supplementary Table 9).

## Discussion

The examination of a large group of patients and caregivers (739 subjects) revealed frequent depressive and anxiety symptoms in both groups. The intensity of depressive symptoms was higher in PWE, mainly in focal epilepsy and recurrent seizures. However, the occurrence and intensity of anxiety symptoms were similar in caregivers and all groups of PWE. Depressive and anxiety symptoms were similarly observed in genetically related and genetically unrelated caregivers, although depressive symptoms tended to be more frequent in genetically related caregivers. The severity of depression in PWE was associated with both anxiety and depression symptoms in their respective caregivers. Unfortunately, suicidal ideation was also identified in both groups, though higher in PWE.

The occurrence of depressive and anxiety symptoms in PWE concurs with previous studies that consistently reported rates of depressive disorders in ~35–44% of PWE (21, 22) of anxiety in nearly 20–40% (23, 24). A correlation between epilepsy outcomes and psychiatric disorders has been previously demonstrated (25, 26). Thus, our results reinforce the hypothesis of common underlying neurobiological mechanisms between these entities (27), as higher frequency and severity of depressive and anxiety symptoms were associated with recurrent seizures. However, the occurrence of these symptoms in caregivers of adult PWE has not been extensively investigated (28), compared to studies performed with caregivers of other chronic diseases such as cancer, Alzheimer's disease, and other neurological disorders (7–9).

Some studies have shown higher parental anxiety and depression levels in children and adolescents with epilepsy (11, 12). Nevertheless, in adults with epilepsy, fewer studies investigated the presence of anxiety, depression (and suicidal ideation) in caregivers (28, 29). In contrast, several studies of caregivers of PWE demonstrated their poor quality of life (12, 30, 31) and increased burden (32). As both anxiety (33) and depression (34) are associated with quality of life, we speculated that the emotional distress identified in the caregivers might be associated with their poor quality of life.

Higher levels of depression (29%) have been described in caregivers of palliative cancer patients (35) and dementia (32%) (8). In our sample, depressive symptoms affected 32% of PWE caregivers, similar to the 33.6% observed in a recent Chinese study with 131 dyads (29). Compared to other diseases, some differences are noteworthy, especially considering the lifetime condition for PWE (especially those with pharmacoresistant seizures), compared to shorter periods of sickness for patients with dementia and cancer. Unfortunately, the impact of epilepsy on family and caregivers has been under-evaluated and mostly neglected (5), compared to several studies performed to recognize and understand both the emotional status and quality of life of caregivers in other chronic medical conditions. These studies have allowed the development of different strategies (36, 37) to improve their emotional status.

The suicidal ideation frequency of 19% in PWE of our sample was higher than the 12.1% prevalence found in a cross-sectional study with 139 patients at North American epilepsy centers (38). A recent meta-analysis of 24 studies showed a pooled suicidal ideation prevalence of 23.2% in PWE (39). Although suicidal ideation was more frequent in PWE (mainly in those with focal epilepsy), it was surprising that the rates (11%) were similar for GGE and caregivers. This proportion is considerably high, compared to rates of suicidal thoughts (0.67%) in the seven days prior to the evaluation of 15,105 Brazilian participants (civil-servants) (40); our observed rate of 11% is closer to the percentage identified by the authors in the subgroup with major depressive disorder (7.7%) (40). This finding is surprising and emphasizes the need for further investigation. In our subjects, the presence of clinical anxiety symptoms was a common critical predictor of suicidal ideation in both PWE and caregivers, which is similar to the results of studies that suggested anxiety as a risk factor for suicidal thoughts (41). As few studies investigated depressive and anxiety symptoms in caregivers of adult PWE, the frequency of suicidal ideation and its predictors also remain poorly recognized and understood in this population.

We identified a similar proportion of anxiety symptoms in caregivers and PWE. Although high levels of anxiety have been repeatedly reported in PWE, the examination of caregivers has received less attention. Interestingly, we observed a similar proportion of symptomatic caregivers (33%) compared to the 31.3% identified in a recent Chinese study (29). One previous study from 1992 examined 44 families and revealed severe anxiety levels in 36.4% of primary caregivers of adult drug-resistant epilepsy (28). Our study's proportion of caregivers with anxiety symptoms was similar to that identified in caregivers of palliative cancer patients (31.2%) (35). This finding, along with the depression rates, raises a concern about the impact of epilepsy on family members and caregivers.

The dyads' analyses showed similar proportions of symptoms of anxiety and depression in caregivers genetically related and unrelated, suggesting the presence of a strong negative environmental impact on caregivers' psychological status. It is essential to highlight that the instruments we used do not allow for diagnosing major depressive disorder (MDD), which may have a bi-directional biological relationship with epilepsy (3, 38). Further studies are necessary to investigate a difference in MDD frequency between caregivers who are genetically related and unrelated to PWE.

Interestingly, the severity of depressive symptoms in PWE is associated with the intensity of anxiety and depressive symptoms in their paired caregivers. These data suggest that the negative impact of epilepsy on caregivers is not negligible and certainly deserves more attention. Although these relationships have been poorly investigated in epilepsy, they have been well recognized in cancer (42) and MDD (43). We observed similar correlations (range 0.25–0.35) to those reported for cancer patient-family caregiver dyads in a Chinese study with 641 dyads (range 0.25–0.32) (44). In 2018, one study reported depressive symptoms in 28.5% of caregivers of 165 people with MDD diagnosis. Multivariate analysis showed that the severity of depressive symptoms in patients with MDD is associated with the severity of depressive symptoms of their caregivers (43).

We observed a similar frequency of concurrent depressive and anxiety symptoms in caregivers (22%) and PWE (27%). This simultaneous finding has been reported in PWE (45) and associated with worse seizure control (46) and reduced quality of life (26). We also have observed this mixed phenomenon in both GGE (25) and mesial temporal lobe epilepsy (47), mainly associated with recurrent seizures. Despite the negative impact on the quality of life, this co-occurrence has not been deeply investigated in caregivers of PWE. Unfortunately, the caregivers of PWE have not received proper attention (5) while facing the lifetime issues of dealing with a chronic, unpredictable disease of their patients. So far, we do not know the best approach to improving their psychological well-being, as dealing with a lifetime condition poses an additional challenge compared to other illnesses. While great effort has been directed to highlight the importance of the treatment of the psychiatric comorbidities of PWE (48) as part of a global approach, our results alert to the need to equal attention to be directed to the caregivers as their emotional distress appears to be equivalent to the PWE and the caregivers of cancer patients. Further studies are required to understand the specific needs of caregivers of PWE, including pharmacological intervention, when necessary. It is also possible that a multidisciplinary treatment for both patients and caregivers, including counseling and support groups, would improve their emotional impairment and quality of life.

### Concurrent Depressive and Anxiety Symptoms in PWE

Our results showed a relationship between depressive and anxiety symptoms in PWE. Those with depressive symptoms were eight times more likely to have anxiety symptoms, and those with anxiety were eight times more likely to have depressive symptoms. As previously reported, this mixture of symptoms is associated with poor seizure control (48). We recently showed that patients with mesial temporal lobe epilepsy with concurrent mood and anxiety disorders were ~4 times more likely to have recurrent seizures than subjects without psychiatric disorders (47). We also observed severe disruption in the functional MRI brain connectivity of GGE patients with mixed anxiety and depressive symptoms (25). The negative impact of this combination on brain function, quality of life (49), and seizure control (48) reinforce the need for better therapies, including pharmacological and non-pharmacological approaches.

### Anxiety, Depressive Symptoms, and Suicidal Ideation in PWE: Relationship With Epilepsy Type and Seizure Control

As previously described, patients with FE (mostly temporal lobe epilepsy) presented more severe depressive symptoms (50) compared to other patients and caregivers. According to our model, patients with FE were two times more likely to present depressive symptoms in comparison to GGE; however, seizure control did not influence the presence of depressive symptoms in this model. Our results differ from a community-based study (with 440 PWE), in which depressive symptoms were equally distributed among different epilepsy types (22). Such discrepancy may be related to our tertiary hospital-based patients and the fact that only 23% of our group with focal epilepsy was free of seizures, while in that study, 56% of all patients were free of seizures for 2 years. This finding reinforces the idea of a bidirectional relationship between temporal lobe epilepsy and depression, as detailed in previous studies (46, 48). On the contrary, anxiety symptoms were similarly observed among the FE, GGE, and caregivers, with equivalent severity. Unlike depression symptoms, subjects with recurrent seizures were two times more likely to present anxiety symptoms, following a previous study that showed an association between anxiety and poorer seizure control (51).

Similar to the analyses of South-Korean patients (74 in the suicide group; 222 patients in the non-suicide group) (52), our multivariate analyses showed the presence of anxiety, frequent seizures, and focal epilepsy associated with the occurrence of suicidal ideation. Suicidal ideation in PWE is complex and multifactorial, including the bidirectional relationship with psychiatric symptoms, exposure to specific anti-seizure drugs, and type of epilepsy syndrome. Some studies have shown an association between suicidal thoughts and increased seizures (53). Unlike what is observed in the general population, we speculate that in epilepsy (with the expected decreasing frequency of seizures over the years) the lower incidence of suicidal ideation at an older age could be related to the strengthening of coping strategies. These approaches are probably developed and consolidated over their lifetime with the restraints of stigma and social and professional limitations (54), added to the clinical aspects of epilepsy that directly impact the patients' quality of life.

## Conclusion

The novelty of our results is mainly associated with the identification of high rates of anxiety, depression and suicidal ideation not only in PWE but also in caregivers. Our findings indicate that specific attention for the emotional health of caregivers is as essential as for PWE. Further studies involving PWE caregivers are required to understand the particular needs and the best approaches, considering the lifetime characteristic of epilepsy for most of the patients.

## Data Availability Statement

The original contributions presented in the study are included in the article/Supplementary Material, further inquiries can be directed to the corresponding author.

## Ethics Statement

The studies involving human participants were reviewed and approved by the Hospital de Clinicas (Unicamp) Research Ethics Committee (CAAE Number: 06816819.5.0000.5404), the research participants were recruited at the Epilepsy Outpatient Clinic of the Hospital de Clínicas of UNICAMP. All subjects included in the sample signed the Informed Consent Form. The patients/participants provided their written informed consent to participate in this study.

## Author Contributions

RJ: designed the study, performed statistical analysis, and wrote the paper. MN: recruited and evaluated subjects, designed the study, performed statistical analysis, and wrote the paper. MM-S: recruited and evaluated subjects, designed the study, and contributed to the discussion session. MA: recruited and evaluated subjects and contributed to the discussion session. SJ and HPe: recruited and evaluated subjects. HPi: supported the statistical analysis. FC: designed the study, contributed to the discussion session, and wrote the paper. CY: recruited and evaluated patients, designed the study, performed statistical analysis, contributed to the discussion session, and wrote the paper. All authors contributed to the article and approved the submitted version.

## Funding

This current study was supported by FAPESP (Grants 2019/11457-8 and CEPID-BRAINN 2013/07559-3) and CAPES (Coordination for the improvement of Higher Education personnel).

## Conflict of Interest

The authors declare that the research was conducted in the absence of any commercial or financial relationships that could be construed as a potential conflict of interest.

## Publisher's Note

All claims expressed in this article are solely those of the authors and do not necessarily represent those of their affiliated organizations, or those of the publisher, the editors and the reviewers. Any product that may be evaluated in this article, or claim that may be made by its manufacturer, is not guaranteed or endorsed by the publisher.
