# Supplementary material for: The Relationship Between Depression and Anxiety Symptoms of Adult PWE and Caregivers in a Tertiary Center
Source: Front Neurol. 2022 Mar 9;13:766009. doi: 10.3389/fneur.2022.766009 (PMC8959587; doi:10.3389/fneur.2022.766009)
Supplement: Supplementary file 1 [file Data_Sheet1.pdf]

Supplementary Material

Supplementary Table 1. Clinical data of PWE according to caregivers' psychiatric groups.

PWE data	Caregivers with depression N = 29 Median [range] or N (%)	Caregivers without depression N = 81 Median [range] or N (%)	**p*-value	Caregivers with anxiety N = 31 Median[range] or N (%)	Caregivers without anxiety N = 74 Median [range] or N (%)	**p*-value	Caregivers with depression plus anxiety N = 16 Median[range] or N (%)	Caregivers without depression plus anxiety N = 84 Median[range] or N (%)	**p*-value
BDI-II scores	22 [3-46]	9 [0-41]	**<0.001**	16 [0-36]	9 [0-41]	**0.041**	22.5 [3-36]	8 [0-46]	**0.007**
BAI scores	18 [1-47]	8 [0-50]	**0.015**	14 [0-39]	8 [0-50]	0.18	17 [1-39]	8.5 [0-50]	0.06
LAEP scores	44.5 [21-68]	37 [19-76]	**0.026**	43 [19-68]	37 [19-76]	0.22	45.5 [26-68]	36.5 [19-76]	**0.038**
Age	29 [6-52]	34 [3-67]	0.28	29 [6-52]	34 [3-67]	0.09	27 [6-52]	34 [3-67]	0.06
Epilepsy duration	15 [1-50]	24 [2-56[	**0.015**	17 [1-50]	24 [2-56]	0.09	23 [2-56]	11.5 [1-50]	**0.03**
Type of epilepsy			0.67			0.34			0.3
Focal	24 (82.8%)	59 (74.7%)		26 (83.9%)	54 (74%)		14 (87.5%)	62 (74.7%)	
Genetic Generalized	3 (10.3%)	11 (13.9%)		4 (12.9%)	10 (13.7%)		3 (12.5%)	11 (13.3%)	
Unknown	2 (6.9%)	9 (11.4%)		1 (3.2%)	9 (12.3%)		0 (0%)	10 (12%)	
Genetic related			0.75			1			1
Yes	22 (75.6%)	57 (70.4%)		22 (71%)	54 (73%)		11 (68.8%)	60 (72.3%)	
No	7 (24.1%)	24 (29.6%)		9 (29%)	20 (27%)		5 (31.3%)	23 (27.7%)	
Seizure control			0.84			0.56			0.5
Recurrent	21 (72.4%)	52 (68.4%)		21 (72.4%)	48 (67.6%)		13 (81.3%)	53 (66.3%)	
Fluctuating	2 (6.9%)	8 (10.5%)		4 (13.8%)	7 (9.9%)		1 (6.3%)	9 (11.3%)	
Seizure-free	6 (20.7%)	16 (21.1%)		4 (13.8%)	16 (22.5%)		2 (12.5%)	18 (22.5%)	

Footnotes Supplementary Table 1: PWE – People with Epilepsy; N – number of caregivers; BDI-II – Beck Depression Inventory-II; BAI – Beck Anxiety Inventory; LAEP – Liverpool Adverse Events Profile; **p*-value for chi-square test of association between categorical variables and for Kruskal-Wallis test of comparison of medians for quantitative variables.

Supplementary Table 2. Sociodemographic and Clinical data (Epilepsy groups and Caregivers), according to epilepsy type.

	FE<br>N = 417<br>Median [range]<br>or N (%)	GGE<br>N = 74<br>Median[range]<br>or N (%)	Caregivers<br>N = 191<br>Median [range]<br>or N (%)	*p-value
Median Age	43 [18-83]	33 [18-60]	47 [18-82]	< 0.001
Gender				
Women	252 (60%)	48 (65%)	137 (72%)	0.026
Men	165 (40%	26 (35%)	54 (28%)	
Employment status				
Unemployment	269 (65%)	38 (51%)	92 (48%)	< 0.001
Employment	148 (35%)	36 (49%)	99 (52%)	
Marital Status				
Married	189 (45%)	20 (27%)	111 (58%)	< 0.001
Non-Married	228 (55%)	54 (73%)	80 (42%)	
Years of Education	11 [0-18]	11 [0-18]	10 [0-18]	0.1
Clinical depression				
N	372	69	174	
Yes	161 (43%)	22 (32%)	56 (32%)	0.026
No	211 (57%)	47 (68%)	118 (68%)	
BDI-II Score	11 [0-57]	9 [0-49]	7 [0-56]	0.018
Suicidal Ideation				
N	404	73	186	
Yes	82 (20%)	8 (11%)	20 (11%)	0.006
No	322 (80%)	65 (89%)	166 (89%)	
Clinical anxiety				
N	376	64	168	
Yes	141 (38%)	23 (36%)	55 (33%)	0.56
No	235 (62%)	41 (64%)	113 (67%)	
BAI Score	10 [0-58]	7 [0-50]	7 [0-51]	0.3
Concurrent clinical depression and anxiety				
N	346	60	160	
Yes	95 (28%)	14 (23%)	35 (22%)	0.38
BDI-II score	24 [14-57]	30 [14-49]	24 [14-56]	0.45
BAI score	25 [14-56]	30 [17-50]	29 [15-51]	0.26
ASD adverse events				
N	371	69	NA	
Yes	100 (27%)	17 (25%)		0.8
No	271 (73%)	52 (75%)		
LAEP score	36 [19-76]	37 [19-68]		0.75

Footnotes Supplementary Table 2: FE – Focal Epilepsy; GGE – Genetic Generalized Epilepsy; BDI-II – Beck Depression Inventory-II; BAI – Beck Anxiety Inventory; ASD – Antiseizure Drugs; LAEP – Liverpool Adverse Events Profile; **p*-value for chi-square test of association between categorical variables and for Kruskal-Wallis test of comparison of medians for quantitative variables.

Supplementary Table 3. Clinical Data of PWE according to the seizure outcome.

	Recurrent seizures N = 332 Median [range] or N (%)	Fluctuating N = 78 Median [range] or N (%)	Seizure-free N = 133 Median [range] or N (%)	*p-value
Median Age	41 [18-73]	40 [18-70]	41 [18-83]	0.6
Gender				
Women	200 (60%)	44 (56%)	82 (62%)	0.75
Men	132 (40%)	34 (44%)	51 (38%)	
Clinical depression				
N	295	74	124	
Yes	140 (48%)	28 (38%)	36 (29%)	0.002
No	155 (52%)	46 (62%)	88 (71%)	
BDI score	13 [0-57]	11 [0-41]	6 [0-47]	< 0.001
Suicidal Ideation				
N	320	77	132	
Yes	81 (25%)	6 (8%)	12 (9%)	< 0.001
No	239 (75%)	71 (92%)	120 (91%)	
Clinical anxiety				
N	292	71	125	
Yes	131 (45%)	20 (28%)	31 (25%)	< 0.001
No	161 (55%)	51 (62%)	94 (75%)	
BAI Score	11 [0-58]	8 [0-54]	5 [0-48]	< 0.001
Concurrent clinical depression and anxiety				
N	270	67	116	
Yes	87 (32%)	15 (22%)	21 (18%)	0.01
BDI-II score	26 [14-57]	21 [14-41]	23 [14-47]	0.37
BAI score	26 [14-56]	25 [14-54]	23 [14-48]	0.35
ASD adverse events				
N	291	73	125	
Yes	98 (34%)	15 (20%)	20 (16%)	< 0.001
No	193 (66%)	58 (80%)	105 (84%)	
LAEP score	39 [19-76]	34 [19-73]	33 [19-73]	< 0.001

Footnotes Supplementary Table 3: BDI-II – Beck Depression Inventory-II; BAI – Beck Anxiety Inventory; ASD – Antiseizure drugs; LAEP – Liverpool Adverse Events Profile; **p*-value for chi-square test of association between categorical variables and for Kruskal-Wallis test of comparison of medians for quantitative variables.

Supplementary Table 4. Variables in the equation for Clinical Depressive Symptoms in PWE.

Factors	B	S.E	Wald	df	Sig.	Exp.(B)	C.I for Exp(B)	
Age	0.001	0.009	0.009	1	0.924	1.001	0.983	1.019
Women	0.505	0.236	4.579	1	0.032	1.657	1.043	2.632
Education (In Years)	0.053	0.035	2.316	1	0.128	1.054	0.985	1.128
Unemployment	0.294	0.239	1.508	1	0.219	1.342	0.839	2.146
Type of Epilepsy			4.411	2	0.110			
Focal	0.761	0.364	4.359	1	0.037	2.139	1.048	4.369
Unknown	0.732	0.476	2.368	1	0.124	2.079	0.818	5.282
Seizure control			2.566	2	0.277			
Recurrent seizures	0.434	0.276	2.459	1	0.117	1.543	0.897	2.653
Fluctuating seizures	0.408	0.373	1.199	1	0.274	1.504	0.724	3.121
Clinical anxiety symptoms	2.082	0.234	79.076	1	< 0.001	8.018	5.067	12.685
Constant	-3.199	0.726	19.415	1	< 0.001	0.041		

Footnotes Supplementary Table 4: PWE – People with Epilepsy; B – Beta; S.E – Standard Error; df – Degrees of Freedom; Exp(B) – Exponential Beta; C.I – Confidence Interval

Supplementary Table 5. Variables in the equation for Suicidal Ideation in PWE.

Factors	B	S.E	Wald	df	Sig.	Exp.(B)	C.I for Exp (B)	
Age	-0.025	0.010	6.197	1	0.013	0.975	0.956	0.995
Women	0.244	0.278	0.770	1	0.380	1.276	0.740	2.200
Unemployment	0.463	0.290	2.546	1	0.111	1.589	0.900	2.808
Type of Epilepsy			5.927	2	0.52			
Focal	1.151	0.492	5.48	1	0.019	3.161	1.206	8.286
Unknown	0.748	0.624	1.437	1	0.231	2.113	0.622	7.179
Seizure control			10.651	2	0.005			
Recurrent seizures	0.944	0.365	6.679	1	0.010	2.570	1.256	5.258
Fluctuating seizures	-0.220	0.587	0.140	1	0.708	0.803	0.254	2.536
Clinical anxiety symptoms	1.655	0.274	36.367	1	< 0.001	5.233	3.056	8.961
Constant	-3.394	0.690	24.198	1	< 0.001	0.034		

Footnotes Supplementary Table 5: PWE – People with Epilepsy; B – Beta; S.E – Standard Error; df – Degrees of Freedom; Exp(B) – Exponential Beta; C.I – Confidence Interval

Supplementary Table 6. Variables in the equation for Clinical Anxiety Symptoms in PWE.

Factors	B	S.E	Wald	df	Sig.	Exp.(B)	C.I for Exp (B)	
Age	0.006	0.009	0.439	1	0.507	1.006	0.988	1.025
Women	0.727	0.248	8.606	1	0.003	2.068	1.273	3.361
Education (In Years)	-0.052	0.035	2.174	1	0.140	0.949	0.886	1.017
Unemployment	0.407	0.251	2.642	1	0.104	1.503	0.920	2.455
Seizure control			7.682	2	0.021			
Recurrent seizures	0.751	0.288	6.788	1	0.009	2.120	1.205	3.730
Fluctuating seizures	0.214	0.398	0.288	1	0.591	1.238	0.567	2.702
Type of epilepsy			1.962	2	0.375			
Focal Epilepsy	-0.401	0.364	1.214	1	0.271	0.670	0.328	1.366
Unknown Epilepsy	-0.007	0.483	< 0.001	1	0.988	0.993	0.385	2.558
Clinical Depressive symptoms	2.084	0.234	79.039	1	< 0.001	8.034	5.075	12.718
Constant	-2.172	0.711	9.338	1	0.002	0.114		

Footnotes Supplementary Table 6: PWE – People with Epilepsy; B – Beta; S.E – Standard Error; df – Degrees of Freedom; Exp(B) – Exponential Beta; C.I – Confidence Interval

Supplementary Table 7. Variables in the equation for combined Clinical Anxiety and Depression Symptoms in PWE.

Factors	B	S.E	Wald	df	Sig.	Exp.(B)	C.I for Exp (B)	
LAEP	2.983	0.291	104.904	1	0.000	19.755	11.162	34.964
Seizure control			1.237	2	0.539			
Recurrent seizures	0.341	0.353	0.931	1	0.335	1.406	0.704	2.811
Fluctuating seizures	0.000	0.510	0.000	1	1.000	1.000	0.368	2.718
Type of epilepsy			0.608	2	0.738			
Focal Epilepsy	0.280	0.460	0.369	1	0.543	1.323	0.537	3.260
Unknown Epilepsy	0.457	0.594	0.593	1	0.441	1.579	0.493	5.054
Unemployment	0.229	0.308	0.551	1	0.458	1.257	0.687	2.300
Women	0.659	0.316	4.353	1	0.037	1.933	1.041	3.590
Familial history of epilepsy	0.165	0.301	0.298	1	0.585	1.179	0.653	2.129
Familial history of psychiatric symptoms	0.298	0.351	0.719	1	0.396	1.347	0.677	2.678
Constant	-3.316	0.596	30.914	1	0.000	0.036		

Footnotes Supplementary Table 7: PWE – People with Epilepsy; LAEP – Liverpool Adverse Events Profile; B – Beta; S.E – Standard Error; df – Degrees of Freedom; Exp(B) – Exponential Beta; C.I – Confidence Interval

Supplementary Table 8. Variables in the equation for Clinical Depressive Symptoms in Caregivers.

Factors	B	S.E	Wald	df	Sig.	Exp.(B)	C.I for Exp (B)	
Age	0.002	0.009	0.039	1	0.844	1.002	0.985	1.019
Women	0.489	0.229	4.547	1	0.033	1.630	1.040	2.555
Marital *Status* (Non-married)	0.072	0.230	0.099	1	0.753	1.075	0.685	1.687
Clinical anxiety symptoms	2.102	0.225	86.963	1	< 0.001	8.181	5.260	12.724
Constant	-1.623	0.431	14.163	1	< 0.001	0.197		

Footnotes Supplementary Table 8: B – Beta; S.E – Standard Error; df – Degrees of Freedom; Exp(B) – Exponential Beta; C.I – Confidence Interval

Supplementary Table 9. Variables in the equation for Anxiety Clinical Symptoms in Caregivers.

Factors	B	S.E	Wald	df	Sig.	Exp.(B)	C.I for Exp (B)	
Age	0.006	0.009	0.391	1	0.532	1.006	0.988	1.024
Women	0.693	0.240	8.327	1	0.004	1.999	1.249	3.201
Education (In Years)	-0.066	0.34	3.748	1	0.053	0.936	0.875	1.001
Marital *Status* (Non-married)	0.502	0.238	4.448	1	0.035	1.652	1.036	2.635
Clinical Depressive symptoms	2.127	0.228	87.281	1	< 0.001	8.389	5.369	13.107
Constant	-1.867	0.622	9.023	1	0.003	0.155		

Footnotes Supplementary Table 9: B – Beta; S.E – Standard Error; df – Degrees of Freedom; Exp(B) – Exponential Beta; C.I – Confidence Interval
